# Incidence and Risk Factors for Surgical Site Infection after Femoral Neck Fracture Surgery: An Observational Cohort Study of 2218 Patients

**DOI:** 10.1155/2022/5456616

**Published:** 2022-06-06

**Authors:** Kexin Zhang, Yunxu Tian, Yan Zhao, Miao Tian, Xiuting Li, Yanbin Zhu

**Affiliations:** ^1^Department of Orthopaedic Surgery, The 3rd Hospital of Hebei Medical University, Shijiazhuang, 050051 Hebei, China; ^2^Hebei Medical University, Shijiazhuang, 050017 Hebei, China; ^3^School of Nursing, Hebei Medical University, Shijiazhuang, 050017 Hebei, China; ^4^Orthopaedic Institution of Hebei Province, Shijiazhuang, 050051 Hebei, China; ^5^Key Laboratory of Biomechanics of Hebei Province, Shijiazhuang, 050051 Hebei, China

## Abstract

**Background:**

Surgical site infection (SSI) was a formidable challenge for surgical management of femoral neck fractures; however, there was a lack of studies with comprehensive variables. We conducted this study to investigate the incidence and risk factors of SSI in elderly patients with femoral neck fractures.

**Methods:**

This was a retrospective study of patients who presented with femoral neck fractures and underwent surgery in our institution between January 2016 and April 2020. All data were collected from a previously validated database. Patients were divided into SSI and non-SSI groups. Univariate and multivariate logistic regression analyses were conducted to identify the risk factors for SSI.

**Results:**

A total of 2218 patients with femoral neck fractures were enrolled in the study, of whom 22 (1%) developed SSI, including 15 (0.7%) superficial and 7 (0.3%) deep SSIs. After multivariable adjustment for confounding factors, patients with and without SSI significantly differ in terms of gender, prolonged time to surgery, CHE < 5 U/L, and injury mechanism.

**Conclusions:**

Our results were helpful for stratification of SSI risk and improved management of hip fracture. Clinicians should be alert to patients with these factors and improve modifiable factors such as preoperative waiting time.

## 1. Introduction

The femoral neck fracture was a common fracture type in orthopedics, especially among elderly individuals, accounting for 48.22% of hip fractures [1, 2]. At present, surgical intervention was the typical treatment modality to help patients with femoral neck fractures restore daily activities as soon as possible [3]. However, surgical site infection (SSI) was one of the most common postoperative adverse outcomes for femoral neck fracture patients, and it was reported that 2.7%-14.9% of hip fracture patients developed SSI after surgery [4–6]. Moreover, postoperative SSI resulted in the doubled risk of mortality and prolonged duration of hospital stay, and the mean financial loss associated with an infection was £7,726 [7, 8]. Therefore, it is necessary to have a clear understanding of the incidence and risk factors of SSI, especially those modifiable, to reduce SSI occurrence.

Published studies had confirmed that age, body mass index (BMI), greater index of comorbidities, hypoalbuminemia, surgical duration, increased duration of anesthesia, current smoking, and elevated fasting blood glucose level were the risk factors of SSI for hip fracture patients [9–12]. However, most previous studies focused on hip fractures, while only a few studies focused on femoral neck fractures separately. In addition, most studies had a small sample size and limited potential factors for adjustment [5]. It is likely that the findings from previous literature are not exactly applicable to patients with femoral neck fractures.

Given that, we incorporated as many factors as possible in the present study to determine the prevalence of SSI and identify the risk factors associated with SSI following surgery of the femoral neck fractures.

## 2. Patients and Methods

### 2.1. Inclusion and Exclusion Criteria

**Figure 1:**
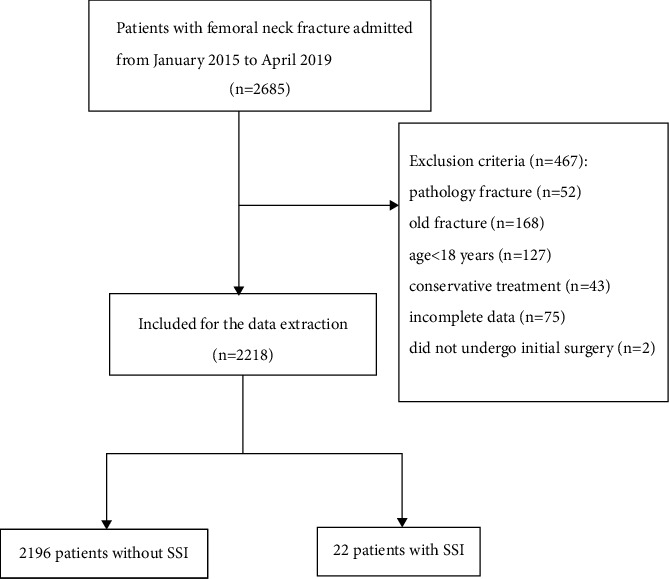
The flow chart for the selection of study participants.

Data on patients who underwent operative management for femoral neck fracture at the 3rd Hospital of Hebei Medical University between January 2016 and April 2020 were extracted from the Surgical Site Infection in Orthopedic Surgery (SSIOS) database. All included patients were 18 years or older and received operative management for an acute closed femoral neck fracture. Patients were excluded if presenting any of the following: old fractures (>21 days), pathological fractures, periprosthetic fractures, not undergoing the initial surgery in our hospital, and with incomplete information (Figure 1). One-year follow-up was conducted after discharge, including the first 1-month follow-up at the outpatient clinic and every 3 months thereafter by telephone or outpatient visit until one year after surgery. The Helsinki Declaration consensus was followed, and the institutional review board approved this research. Because no patient identity information was included, the requirement for informed consent was waived.

### 2.2. Definition of SSI

According to the diagnosis criteria of the Center for Disease Control (CDC), SSI was diagnosed and classified into two types [13]. Superficial SSI was defined as an infection involving the skin and subcutaneous tissue of the surgical site, with wound signs and symptoms (swelling, redness, pain, and heat) and was often resolved by physiotherapy or oral antibiotics. Deep SSI was defined as an infection involving the fascia, muscle, and deep soft tissues and was diagnosed based on at least one of the following: fascia or muscle infections, persistent wound discharge or dehiscence, visible abscess or gangrene requiring surgical debridement, and implant removal or exchange.

### 2.3. Prophylaxis

According to the Surgical Site Infection Guidelines developed by the American College of Surgeons and Surgical Infection Society, prophylactic antibiotics were administered for SSI prevention [14]. Based on the hospital policy, 1 to 3 grams of cefazolin were intravenously used within 0.5 hours before the incision and 24 hours postoperatively.

### 2.4. Data Collection of Variables

We collected 63 variables from demographic variables, fracture-related variables, operation-related variables, and preoperative laboratory parameters. Demographic variables included gender, age, body mass index (BMI), residential location (rural or urban), and comorbidities (diabetes, hypertension, cardiovascular disease, cerebrovascular disease, and so on). Fracture-related variables included injury mechanism (low or high energy), preoperative duration, and concurrent fractures. Operation-related variables included reduction methods (closed or open reduction), American Society of Anesthesiologists grade (ASA), anesthesia pattern, operative time, fixation type, intraoperative blood loss, and intraoperative blood transfusion. Preoperative laboratory parameters included platelet (PLT), albumin/globulin (A/G), alanine transaminase (ALT), white blood cells (WBC), red blood cell (RBC), albumin (ALB), lymphocytes (LYM), very low-density lipoprotein (VLDL), mean corpuscular volume (MCV), uric acid (UA), hemoglobin (HGB), hypersensitive C-reactive protein (HCRP), platelet distribution width (PDW), glucose (GLU), total protein (TP), and globulin (GLOB).

### 2.5. Statistical Analysis

Continuous variables were described as mean and standard deviation ($\bar{x}\pm \text{SD}$), while categorical variables were expressed as frequency and percentage. The receiver operating characteristic (ROC) analysis was used for quantitative data such as time to surgery and operative time to determine the optimum cut-off values. The Shapiro-Wilk test was used to test continuous variables for normal distribution. According to the distribution pattern, Student's *t-*test or the Mann-Whitney *U* test was performed. The Pearson chi-square test or Fisher exact test was applied to compare the categorical variables. Univariate and multivariate logistic regression analyses determine the independent risk factors. *P* < 0.05 indicated statistical significance. Model fit was evaluated by the Hosmer-Lemeshow goodness-of-fit test, and acceptable fitness was accepted when *P* was <0.05. The SPSS 26.0 software was used for data analysis, and GraphPad Prism 9 software was used for mapping.

## 3. Result

A total of 2218 patients with femoral neck fractures were collected in the current study. There were 22 (1%) patients who developed SSI, and 2196 (99%) patients were without SSI. The rate of superficial infection was 0.7% while it was 0.3% for the deep SSI.

**Figure 2:**
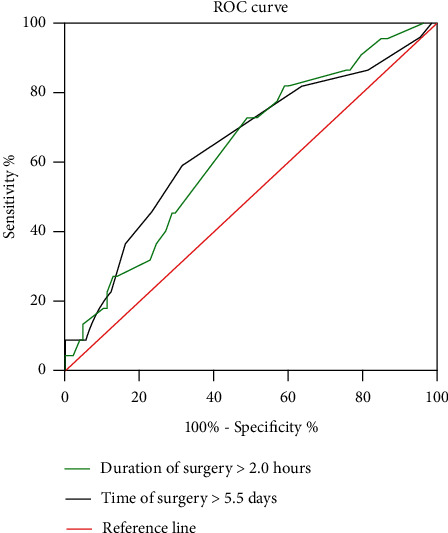
The optimum cut-off value of some continuous variables associated with SSI was detected by ROC analysis.

The ROC analysis showed the optimum cut-off value for time to surgery, and operative time was 5.5 days (considering the clinical practice, we use 6 days) and 2 hours, respectively (Table 1 and Figure 2). The univariate analysis showed that concurrent fractures (*P* = 0.01), injury mechanism (high energy) (*P* = 0.001), preoperative waiting time ≥ 6 days (*P* = 0.006), operative time > 2 hours (*P* ≤ 0.001), gender (male) (*P* = 0.001), ALB < 35 g/L (P =0.035), DBIL >6 *μ*mol/L (P =0.017), and CHE < 5 U/L (*P* = 0.02) were identified as significant risk factors for the development of SSI and to be entered into the multivariate logistic regression analysis (Table 2). The result of multivariate analysis showed gender (male vs. female) (OR, 3.521, *P* = 0.010), preoperative waiting time ≥ 6 days (OR, 2.85, *P* = 0.019), CHE < 5 U/L (OR, 2.861, *P* = 0.018), and injury mechanism (high energy) (OR, 3.688, *P* = 0.005) were independent risk factors (Table 3). The multivariate predictive model was adequate (Hosmer-Lemeshow test, *X*^2^ = 3.326, *P* = 0.650).

## 4. Discussion

There is still lake of some large-sample studies on the incidence and risk factors of SSI in patients with femoral neck fractures. In our study, we used a previously validated database to resolve this issue. We found the SSI rate was 1%. Univariate and multivariate analyses revealed that gender (male), preoperative waiting time ≥ 6 days, CHE < 5 U/L, and injury mechanism (high energy) were independent risk factors for SSI occurrence.

In this study, the incidence of SSI was 1%, remarkably lower than those (2–7%) reported in other literature [12, 15–18]. The possible reasons could be various, involving heterogeneous patient characteristics, surgical interventions, study designs, and follow-up period. Slobogean et al. [19] found the incidence rate of SSI was 5.1% in young femoral neck fracture patients (<60 years old). A recent prospective observational study by Ji et al. reported the rate of 3.76% [15]. And this was probably due to the following reason that our cohort included patients at a relatively older age (66.8 vs. >60 years in Slobogean et al. [19]) and was a retrospective study (vs. prospective study in Ji et al. [15]). In addition, the bacterial culture results were used for infection diagnosis in some studies [20, 21], which might also affect the incidence of SSI.

Gender proved to be a risk factor associated with SSI in the current study, consistent with the findings of most studies [22–24]. In this study, males were 3.2-fold more susceptible than females to SSI (OR, 3.214, *P* = 0.019), which was consistent with the finding by Zhao et al. [24]. However, this factor was not conclusive, because some scholars found no significant correlation between gender and SSI [4]. The possible underlying mechanism may be the higher prevalence with male population, such as tobacco and alcohol, which were well-established factors to influence the occurrence of SSI [25–29]. It was also likely that other factors that were more prevalent in males, such as higher injury mechanism in relatively younger patients, contributed to this observed risk [30–32].

Preoperative waiting time as a risk factor for SSI was a hot-discussed point, and its relation to a range of adverse outcomes has been reported [33–35]. Current clinical practice guidelines recommend surgical treatment within 24-48 hours [36]. However, the cut-off time for preoperative waiting time applied in clinical practice varied [37]. Chen et al. [38] conducted an observational study of 889 hip fracture patients and found patients with the preoperative waiting time ≥ 24 h exhibited a higher risk of complications. In another prospective study of 1941 patients with intertrochanteric fracture, time to surgery > 4 days was reported as a significant risk factor for SSI [24]. Our institution as a tertiary referral and orthopedic-specified hospital, received a substantial proportion of patients who were transferred from secondary hospitals, presenting with severer fractures or more complex medical conditions. Thus, a long time would be needed to adjust the patients' preoperative physical status to improve the tolerance to operation. Furthermore, during the past ten years, the number of annual orthopedic surgeries in our hospital had increased to approximately 50,000 cases per year, which made it very tense for the operative arrangement, more during the past 3 years since COVID-19 become epidemic. Over 2/3 of patients had to be operated without the recommended early operation time frame (within 24-48 h of injury) [39–41]. The prolonged preoperative waiting time may be attributable to the physiologically unstable state of patients and insufficient staff and equipment. Thus, surgeons should be aware of the effect of preoperative waiting time on SSI and provide adequately targeted preoperative interventions and optimized operative procedures.

To our knowledge, this study was the first to identify CHE as an independent risk factor for SSI, and the relation magnitude was 2.86. The underlying pathophysiologic mechanism may be various, including but not limited to trauma per se [36, 37]. For example, Ba et al. [42] showed the systemic inflammatory response following trauma reduced the liver synthesis of CHE, and the greater the decrease in amplitude of CHE suggested a more severe trauma accompanied by serious soft tissue damage [43], which could provide a fine environment for bacterial colonization and infection. At the same time, a low CHE value could indicate malnutrition which also had detrimental effects on wound healing [5, 44–47]. Anyway, as an important indicator to evaluate the risk of SSI, CHE should be recognized and kept in mind in practice to alert the possibility of SSI.

The injury mechanism (high energy) has been verified as an independent risk factor of SSI [48], which was reconfirmed in the present study. First, the high-energy injuries meant the resultant more damaged skin, the fractured bone itself, and even open wounds, which allowed microbial easy to colonize and contaminate to give rise to SSI [43, 49]. Moreover, our data showed that the operative time in the high-energy group was approximately twice that of the low-energy group, which might have prolonged the exposure of wounds to the air, resulting in the increased risk of infection [50]. Therefore, the mechanism of injury should be meticulously inquired for a comprehensive evaluation, and necessary debridement procedures should be immediately carried out, even in some cases seemingly not serious.

The present study had several strengths, including a large-sample study, adjustment for comprehensive and numerous variables covering various aspects, and ROC analysis used for detecting highly sensitive cut-off values for some continuous variables. This study was not without limitations. The retrospective design inherited the selective bias. Although a multivariate regression model was used to minimize numerous confounding variables as much as possible, unmeasured or unknown confounders remain. And our results might be less generalizable to institutions, especially in developed countries, which strictly implement the early operation (within 24-48 h) strategy.

## 5. Conclusion

In summary, the overall incidence of SSI for femoral neck fracture following operative treatment was 1%. Gender (male), preoperative waiting time ≥ 24 h, CHE < 5 U/L, and injury mechanism (high energy) were independent risk factors for the SSI after femoral neck fracture surgery. Orthopedists should take these risk factors into consideration when evaluating and stratifying the risk of SSI following surgeries of hip fracture.

## Figures and Tables

**Table 1:** Optimum cut-off value of continuous variables detected by the ROC analysis.

Variables	Cut-off value	Area under the ROC curve (AUC)	*P* value	95% CI
Time to surgery	5.5	0.65	0.02	0.527-0.771
Operative time	2.0	0.66	0.03	0.526-0.746

Abbreviations: CI: confidence interval; ROC: receiver operating characteristic.

**Table 2:** Univariable analyses of risk and prognostic factors.

Variables	Total patients (*N* = 2218)	Non-SSI (*N* = 2196)	SSI (*N* = 22)	*P* value
Intraoperative blood loss (mL), *n* (%)				0.198
≤200	1151 (51.9)	1143 (52.0)	8 (36.4)	
201-400	716 (32.3)	708 (32.2)	8 (36.4)	
401-600	231 (10.4)	229 (10.4)	2 (9.1)	
601-800	63 (2.8)	62 (2.8)	1 (4.5)	
801-1000	26 (1.2)	24 (1.1)	2 (9.1)	
>1000	31 (1.4)	30 (1.4)	1 (4.5)	
Age (years), mean (SD)	66.8 ± 15.5	63.4 ± 13.7	66.9 ± 15.6	0.134
Hypertension, *n* (%)	854 (38.5)	848 (38.6)	6 (27.3)	0.277
Diabetes, *n* (%)	369 (16.6)	366 (16.7)	3 (13.6)	0.927
Cerebrovascular disease, *n* (%)	500 (22.5)	495 (22.5)	5 (22.7)	1.000
Cardiovascular disease, *n* (%)	459 (20.7)	453 (20.6)	6 (27.3)	0.616
Concurrent fractures, *n* (%)	262 (11.8)	255 (11.6)	7 (31.8)	0.010^∗^
Tumour, *n* (%)	33 (1.5)	32 (1.5)	1 (4.5)	0.760
Renal disease, *n* (%)	64 (2.9)	64 (2.9)	0 (0)	0.863
Urinary tract infection, *n* (%)	14 (0.6)	14 (0.6)	0 (0)	1.000
Residential location (urban), *n* (%)	984 (44.36)	973 (44.3)	11 (50)	0.861
Injury mechanism (high energy), *n* (%)	254 (11.45)	246 (11.2)	8 (36.4)	0.001^∗^
Reduction methods (open reduction), *n* (%)	44 (2.0)	42 (1.9)	2 (9.1)	0.069
Preoperative waiting time (≥6 days), *n* (%)	703 (31.7)	690 (31.4)	13 (59.1)	0.006^∗^
Type of anesthesia (general), *n* (%)	1009 (45.5)	999 (45.5)	10 (45.5)	0.997
Side (left), *n* (%)	1174 (53.46)	1166 (53.1)	8 (36.4)	0.118
Fixation type, *n* (%)				1.000
Internal fixation	1846 (83.2)	1828 (83.2)	18 (81.8)	
External fixation	372 (16.8)	368 (16.8)	4 (18.2)	
Operative time (>2.0 hours), *n* (%)	1088 (49.1)	1072 (48.8)	16 (72.7)	0.026^∗^
ASA ≥ 3, *n* (%)	803 (36.2)	794 (36.2)	9 (40.9)	0.644
Gender (male), *n* (%)	853 (38.5)	837 (38.1)	16 (72.7)	0.001^∗^
BMI, *n* (%)				0.072
<18.5	56 (2.5)	54 (2.5)	2 (9.1)	
18.5-23.9	1300 (58.6)	1285 (58.5)	15 (68.2)	
24-27.9	565 (25.5)	560 (25.5)	5 (22.7)	
28-31.9	280 (12.6)	280 (12.8)	0 (0.0)	
≥32	17 (0.8)	17 (0.8)	0 (0.0)	
Tp < 65 g/L, *n* (%)	1448 (65.3)	1432 (65.2)	16 (72.7)	0.461
ALB < 35 g/L, *n* (%)	1017 (45.9)	1002 (45.6)	15 (68.2)	0.035^∗^
GLOB (references 20-40 g/L), *n* (%)				0.459
<20	300 (13.5)	295 (13.4)	5 (22.7)	0.167
>40	10 (0.5)	10 (0.5)	0 (0.0)	0.905
A/G (references 1.2-2.4), *n* (%)				0.242
<1.2	370 (16.7)	364 (16.6)	6 (27.3)	
>2.4	30 (1.4)	29 (1.3)	1 (4.5)	
ALT (references 9-50 U/L), *n* (%)				0.738
<9	193 (8.7)	192 (8.7)	1 (4.5)	0.417
>50	171 (7.7)	169 (7.7)	2 (9.1)	0.515
AST (references 15-40 U/L), n (%)				0.058
<15	463 (20.9)	462 (21.0)	1 (4.5)	0.068
>40	214 (9.6)	210 (9.6)	4 (18.2)	0.156
TBIL (>26 *μ*mol/L), *n* (%)	186 (8.4)	182 (8.3)	4 (18.2)	0.201
DBIL (>6 *μ*mol/L), *n* (%)	688 (31.0)	676 (30.8)	12 (54.5)	0.017^∗^
IBIL (>14 *μ*mol/L), *n* (%)	230 (10.4)	227 (10.3)	3 (13.6)	0.878
ALP (references 45-125 U/L), *n* (%)				0.099
<45	266 (12.0)	263 (12.0)	3 (13.6)	
>125	71 (3.2)	68 (3.1)	3 (13.6)	
GGT (references 10-60 U/L), *n* (%)				0.527
<10	83 (3.7)	81 (3.7)	2 (9.1)	
>60	210 (9.5)	208 (9.5)	2 (9.1)	
CHE (references 5-12 U/L), *n* (%)				0.020^∗^
<5	436 (19.7)	426 (19.4)	10 (45.5)	≤0.001^∗^
>12	21 (0.9)	21 (1.0)	0 (0.0)	0.818
TBA (references 1-10 *μ*mol/L), *n* (%)				0.064
<1	284 (12.8)	277 (12.6)	7 (31.8)	
>10	164 (7.4)	163 (7.4)	1 (4.5)	
HCRP (>8), *n* (%)	1818 (82)	1802 (82.1)	16 (72.7)	0.393
CK (>), *n* (%)	716 (32.3)	708 (32.2)	8 (36.4)	0.681
CKMB (>), *n* (%)	163 (7.3)	160 (7.3)	3 (13.6)	0.468
LDH (>), *n* (%)	638 (28.8)	629 (28.6)	9 (40.9)	0.206
HBDH (>), *n* (%)	618 (27.9)	614 (28.0)	4 (18.2)	0.309
TC (>), *n* (%)	296 (13.3)	294 (13.4)	2 (9.1)	0.784
TG (>), *n* (%)	186 (8.4)	183 (8.3)	3 (13.6)	0.613
Sodium (references 137-147 mmol/L), *n* (%)				0.131
<137	851 (38.4)	838 (38.2)	13 (59.1)	
>147	8 (0.4)	8 (0.4)	0 (0.0)	
K (references 3.5-5.3 mmol/L), *n* (%)				0.736
<3.5	247 (11.1)	245 (11.2)	2 (9.1)	0.549
>5.3	25 (1.1)	25 (1.1)	0 (0.0)	0.778
CL (references 99-110 mmol/L), *n* (%)				0.400
<99	295 (13.3)	291 (13.3)	4 (18.2)	0.334
>110	84 (3.8)	82 (3.7)	2 (9.1)	0.202
TCO_2_ (references 20–30 mmol/L), n (%)				0.263
<20	101 (4.6)	99 (4.5)	2 (9.1)	0.265
>30	95 (4.3)	95 (4.3)	0 (0.0)	0.380
GLU (>6.1), *n* (%)	982 (44.3)	974 (44.4)	8 (36.4)	0.453
UREA (>8), *n* (%)	322 (14.5)	319 (14.5)	3 (13.6)	1.000
CREA (references 57-97 mmol/L), *n* (%)				0.242
<57	1003 (45.2)	994 (45.3)	9 (40.9)	0.426
>97	118 (5.3)	118 (5.4)	0 (0.0)	0.376
UA (references 208-428 mmol/L), *n* (%)				0.446
<208	939 (42.3)	932 (42.4)	7 (31.8)	0.316
>428	98 (4.4)	96 (4.4)	2 (9.1)	0.254
WBC (references 3.5-9.5 10^9^/L), *n* (%)				0.825
<3.5	14 (0.6)	14 (0.6)	0 (0.0)	0.869
>9.5	773 (34.9)	766 (34.9)	7 (31.8)	0.764
NEU (references 1.8-6.3 10^9/^L), *n* (%)				0.516
<1.8	9 (0.4)	9 (0.4)	0 (0.0)	0.914
>6.3	1163 (52.4)	1154 (52.6)	9 (40.9)	0.277
LYM (references 1.1-3.2 10^9^/L), *n* (%)				0.326
<1.1	1037 (46.8)	1030 (46.9)	7 (31.8)	0.158
>3.2	15 (0.7)	15 (0.7)	0 (0.0)	0.861
MON (references 0.1-0.6 10^9^/L), *n* (%)				0.865
<0.1	0 (0.0)	0 (0.0)	0 (0.0)	—
>0.6	1271 (57.3)	1258 (57.3)	13 (59.1)	0.865
EOS (references 0.02-0.52 10^9^/L), *n* (%)				0.077
<0.02	686 (30.9)	684 (31.1)	2 (9.1)	
>0.52	10 (0.5)	10 (0.5)	0 (0.0)	
BAS (>0.06), *n* (%)	167 (7.5)	163 (7.4)	4 (18.2)	0.134
RBC (>5.8), *n* (%)	2 (0.1)	2 (0.10)	0 (0.00)	1.000
HGB (<110/120), *n* (%)	843 (38)	833 (37.9)	10 (45.5)	0.470
HCT (references 40%-50%), *n* (%)				0.530
<40	1781 (80.3)	1765 (80.4)	16 (72.7)	0.255
>50	437 (19.7)	431 (19.6)	6 (27.3)	0.255
MCV (references 82-100 fL), *n* (%)				0.221
<82	67 (3.0)	66 (3.0)	1 (4.5)	0.492
>100	141 (6.4)	141 (6.4)	0 (0.0)	0.234
MCH (references 22-34 pg), *n* (%)				0.247
<27	66 (3.0)	65 (3.0)	1 (4.5)	0.487
>34	130 (5.9)	130 (5.9)	0 (0.00)	0.263
MCHC (references 316-354 g/L), *n* (%)				0.237
<316	45 (2.0)	45 (2.0)	0 (0.00)	0.636
>354	95 (4.3)	95 (4.3)	0 (0.00)	0.380
PLT (references 125-350 10^9^/L), *n* (%)				0.735
<125	165 (7.4)	164 (7.5)	1 (4.5)	0.504
>250	572 (25.8)	565 (25.7)	7 (31.8)	0.516
MPV (references 7.4-11.0 fL), *n* (%)				0.265
<7.4	295 (13.3)	290 (13.2)	5 (22.7)	0.158
>11.0	66 (3.0)	66 (3.0)	0 (0.00)	0.513

ASA: American Society of Anesthesiologists grade; BMI: body mass index; TP: total protein; ALB: albumin; GLOB: globulin; A/G: albumin/globulin; ALT: alanine transaminase; AST: aspartate aminotransferase; TBIL: total bilirubin; DBIL: direct bilirubin; IBIL: indirect bilirubin; ALP: alkaline phosphatase; GGT: *γ*-glutamyl transpeptidase; CHE: cholinesterase; TBA: total bile acid; HCRP: hypersensitive C-reactive protein; CK: creatine kinase; CKMB: creatine kinase isoenzyme; LDH: lactic dehydrogenase; HBDH: hydroxybutyrate dehydrogenase; TC: total cholesterol; TG: triglyceride; GLU: glucose; UREA: serum urea; CREA: creatinine; UA: uric acid; WBC: white blood cell; NEU: neutrophile; LYM: lymphocyte; MON: mononuclear cell; EOS: eosinophilic granulocyte; BAS: basophilic granulocyte; RBC: red blood cell; HGB: hemoglobin; HCT: hematocrit; MCV: mean corpuscular volume; MCH: mean corpuscular hemoglobin; MCHC: mean corpuscular hemoglobin concentration; PLT: platelet; MPV: mean platelet volume. ^∗^Significant variables.

**Table 3:** OR, 95% CI, and *P* value for independent risk factors in the multivariate logistic regression analysis of SSI.

Variables	OR	95% CI (lower limit)	95% CI (upper limit)	*P*
Gender (male)	3.521	1.345	9.218	0.010
Preoperative waiting time (≥6 days)	2.850	1.189	6.831	0.019
CHE (<5 U/L)	2.861	1.200	6.820	0.018
Injury mechanism (high energy)	3.688	1.492	9.117	0.005

CHE: cholinesterase. ^∗^Significant variables.

## Data Availability

Data is available from the authors upon request.
